# Quality improvement studies in nursing homes: a scoping review

**DOI:** 10.1186/s12913-021-06803-8

**Published:** 2021-08-12

**Authors:** Mark Toles, Cathleen Colón-Emeric, Elizabeth Moreton, Lauren Frey, Jennifer Leeman

**Affiliations:** 1 University of North Carolina at Chapel Hill, Chapel Hill, USA; 2 Duke University and Durham VA GRECC, Durham, USA

**Keywords:** Quality improvement, Nursing homes, Long term care, Residential aged care, Implementation strategies

## Abstract

**Background:**

Quality improvement (QI) is used in nursing homes (NH) to implement and sustain improvements in patient outcomes. Little is known about how QI strategies are used in NHs. This lack of information is a barrier to replicating successful strategies. Guided by the Framework for Implementation Research, the purpose of this study was to map-out the use, evaluation, and reporting of QI strategies in NHs.

**Methods:**

This scoping review was completed to identify reports published between July 2003 through February 2019. Two reviewers screened articles and included those with (1) the term “quality improvement” to describe their methods, or reported use of a QI model (e.g., Six Sigma) or strategy (e.g., process mapping) (2), findings related to impact on service and/or resident outcomes, and (3) two or more NHs included. Reviewers extracted data on study design, setting, population, problem, solution to address problem, QI strategies, and outcomes (implementation, service, and resident). Vote counting and narrative synthesis were used to describe the use of QI strategies, implementation outcomes, and service and/or resident outcomes.

**Results:**

Of 2302 articles identified, the full text of 77 articles reporting on 59 studies were included. Studies focused on 23 clinical problems, most commonly pressure ulcers, falls, and pain. Studies used an average of 6 to 7 QI strategies. The rate that strategies were used varied substantially, e.g., the rate of in-person training (55%) was more than twice the rate of plan-do-study-act cycles (20%). On average, studies assessed two implementation outcomes; the rate these outcomes were used varied widely, with 37% reporting on staff perceptions (e.g., feasibility) of solutions or QI strategies vs. 8% reporting on fidelity and sustainment. Most studies (*n* = 49) reported service outcomes and over half (*n* = 34) reported resident outcomes. In studies with statistical tests of improvement, service outcomes improved more often than resident outcomes.

**Conclusions:**

This study maps-out the scope of published, peer-reviewed studies of QI in NHs. The findings suggest preliminary guidance for future studies designed to promote the replication and synthesis of promising solutions. The findings also suggest strategies to refine procedures for more effective improvement work in NHs.

**Supplementary Information:**

The online version contains supplementary material available at 10.1186/s12913-021-06803-8.

## Background

In the U.S., staff in 15,600 nursing homes (NH) care for about 1.3 million older adults each day [1]. In addition to providing housing, three meals a day, and personal care, NHs also provide skilled nursing care, 24-h supervision, and rehabilitation services, such as physical therapy [2]. Frailty and serious illnesses are common in NHs, where 50% of older adults have dementia and more than 90% require assistance with bathing and other activities of daily living [1, 3]. Ensuring high quality care for NH residents continues to be a major challenge [4]. Factors contributing to this challenge include high NH staff turnover, fragmented communication internal and external to NHs, limited resources to pay for clinical staff and technology tools, and the training and education of staff. Owing to these challenges, improving the quality of care of NH residents remains a high priority [5–7].

Government regulations and alternative payment models have been important drivers of improved quality in NHs [8]. In 1987 the Nursing Home Reform Act mandated resident-level care planning in NHs and comprehensive inspection of NHs every 15 months [9]. In the early 2000s, market-based reforms, such as public-reporting of NH quality, were implemented to generate demand for NHs with higher publicly-reported quality indicators [10, 11]. External standards and incentives have contributed to the improvement of quality of care [12–14]; however, they are not sufficient to remedy persisting NH quality challenges, which include fall prevention, dementia care, antibiotic stewardship, and preventing avoidable hospitalizations, among others.

To address quality challenges, NH leaders and researchers use a range of quality improvement tools, methods, and strategies (hereafter referred to as “QI strategies”) to evaluate the quality of care, identify local causes of quality deficits, and implement or sustain improvements in care [15–17]. Starting in 2014, the U.S. Centers for Medicare and Medicaid Services mandated that all NHs establish Quality Assurance and Performance Improvement (QAPI) programs as a requisite for receiving federal funding. However, little is known about how QI strategies are used in NHs, their effectiveness, or how to replicate or apply proven strategies across settings [18]. The large majority of evidence from QAPI programs and other QI work in NHs is not published. Prior reviews described a range of clinical problems that were addressed, such as patient falls, and the use of improvement strategies to support changes in clinical care [19–21]. However, these reviews are now 6–15 years old and omit details on the types of QI strategies that were used and the implementation outcomes measured. We address these limitations by synthesizing evidence across QI studies in NHs, thereby informing the design of future QI studies. Synthesizing evidence from QI studies is difficult due to variations in terminology, outcomes measurement, and how findings are reported across methodologies [21]. Thus, in this review, we adapted Proctor and colleagues’ widely-used “Framework for Implementation Research” as a guide for mapping the literature on QI strategies in NHs [22].

**Fig. 1:**
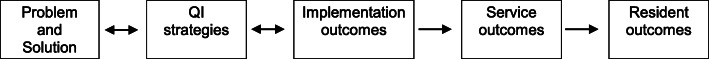
Adaptation of the Framework for Implementation Research [22]

The Framework for Implementation Research describes the pathway from clinical interventions, to implementation strategies, and then to service (e.g., safety and equity) and client outcomes [22]. As illustrated in Fig. 1, our adaptation of the framework more broadly defines domains in the framework for our focus on QI in NHs. In contrast to implementation research, which begins with the domain of evidence-based interventions, QI often begins with a problem and then transitions to one or more solutions to address the problem; these solutions may or may not be evidence-based interventions [23, 24]. Therefore, the first domain in our adaptation of Proctor’s framework includes the problem and the solution(s).

In the second domain we replace “implementation strategies with “QI strategies.” This domain includes strategies that are applied to understand the problem, ascertain the fit of solutions to address the problem, and integrate those solutions into routine practice. Often referred to as tools, interventions, or methods, examples of QI strategies include root cause analysis, Plan-Do-Study-Act (PDSA) cycles, and others [25]. In most QI models (e.g., the Improvement Model), QI strategies are designed to engage local providers and staff and walk them through a systematic, multi-step approach to developing “fit-for-purpose solutions.” [26] The final three domains in the framework are three types of outcomes. These include “implementation outcomes”, which assess the impact of QI strategies on factors that determine the successful integration of a solution into routine practice. For example, “adoption” is an implementation outcome defined as the extent to which a solution is initiated by settings and providers [27, 28]. “Service outcomes” assess the quality of services, with quality encompassing efficiency, safety, effectiveness, equity, patient-centeredness, and timeliness [28]. The adapted framework culminates in changes in “resident outcomes” [22]; in other words, changes in the health and wellbeing of NH residents.

Applying this adapted framework, the purpose of this study was to conduct a scoping review of published literature on QI in NHs. The intent of the review was to map-out how studies were using, evaluating, and reporting QI strategies and outcomes.

## Methods

We conducted a scoping review with the goal of mapping the heterogeneity of study designs, QI approaches, and outcome measures rather than synthesizing findings on the effectiveness of specific strategies. We followed the PRISMA-ScR (Preferred Reporting Items for Systematic Reviews and Meta-Analysis extension for Scoping Reviews) [29].

### Data sources and searches

We collaborated with a health sciences librarian and conducted a systematic literature search to identify articles relating to QI in NHs. We searched PubMed, CINAHL Plus with Full Text (EBSCO), and Embase for English language articles published between July 1, 2003 through February 28, 2019. We searched for keywords and Medical Subject Headings related to NHs, assisted living facilities, housing for the elderly, skilled nursing facilities, or residential facilities, as well as keywords and subjects related to quality assurance, quality improvement, performance improvement, and Lean and Six Sigma. The full search is included in Additional File 1. Preset database filters were used to exclude non-research articles, such as conference abstracts, editorials, letters, or dissertations. The results were combined in EndNote and duplicate reports were removed before beginning the title/abstract screening in Covidence [30].

### Study selection

Two reviewers (MT and JL) independently screened the titles and abstracts of 2069 articles from the initial search and 233 from the update (a total of 2302 articles). Discrepancies in the selection of articles to include were resolved by consensus. Articles were included if they were empirical studies reporting on QI projects or research studies conducted in NHs. The inclusion criteria were (1) peer-reviewed articles published in the English language between July 2003 and February 2019 (2), used the term “quality improvement” to describe their methods or reported using a quality improvement model (e.g., Six Sigma) or strategy (e.g., process mapping, PDSA) and (3) reported findings related to impact on either service and/or resident outcomes. We excluded articles that reported findings from only one NH as they generally are case reports with limited potential to contribute to generalizable knowledge about QI strategies [15].

### Data charting process

Three reviewers (MT, JL, LF), working in pairs, reviewed the full text of included articles and used a standardized template to extract data. During the extraction process, we noted when authors referred to additional articles on their studies and added these articles to the review. The adapted version of the Framework for Implementation Research guided development of the data extraction template. As summarized in Table 1 and below, the research team drew on both the QI and implementation science literature to develop the terminology and definitions for data extracted. Data were extracted on study design, study setting and population, problem targeted, solution selected to address problem, QI strategies used, and outcomes (implementation, service, and resident). We extracted descriptions of the solutions to address the targeted problem, and in cases where the solution was an intervention, we extracted the intervention name, if available. We applied an iterative process to code QI strategies and implementation outcomes. We developed an initial coding strategy, derived from existing taxonomies and lists of QI and implementation strategies [22, 31, 32] as well as implementation outcomes [22, 33]. We then applied and iteratively revised the coding strategy to fully capture data identified in our review.

**Table 1:** Terminology and definitions for data extracted

Domain	Construct	Definition
Problem/ Solution	Problem	The gap in NH care and/or patient outcomes that authors targeted for improvement
	Solution	The approach selected to address a problem, defined broadly to include both systems level changes to improve the quality of care delivery and clinical intervention programs, practice guidelines, policies and procedures [58].
	Project name	The name authors provided for the study or project to address a problem
QI Strategies	Site champion	Designate an individual who will promote and support an initiative [31]
	QI or implementation team	Teams that were establish and supported to plan and guide implementation [31]
	Technical assistance	Interactive support that is individualized to the specific needs of individuals or teams [59]
	Training: in-person or virtual	Educational and/or skill-building sessions [59]
	Tools/Toolkits	Electronic or print resource used to plan, deliver, implement, or evaluate a solution [59]
	Process mapping	Methods used to visually represent the way a care process works, referred to as a process map or flow chart [17, 60]
	Root cause analysis	Methods used to gain diverse perspectives on factors contributing to a problem. Includes Ishikawa or fishbone diagram and the five why’s exercise, among others [17]
	Audit and feedback	Methods used to collect and summarize performance data and report it those implementing a solution [31]
	Plan-do-study-act cycles (PDSA)	A multistep, rapid, and cyclical process for assessing whether a change led to improvement [17]
	Quality monitoring systems	Systems and procedures that are developed to monitor care delivery and/or outcomes for the purpose of quality improvement [31]
	Health record modifications	Change the health record to support implementation of the solution [31]
	Learning collaborative	Bringing together staff and providers from multiple organizations to foster a “collaborative learning environment” [31]
Implementation outcomes	Adoption	Proportion of NHs invited that agree to participate in a QI initiative” [33]
	Reach to staff	Number and/or proportion of eligible staff who participate in a QI initiative [33]
	Reach to residents	Number and/or proportion of eligible NH residents who received or were exposed to a solution [33]
	Fidelity	The degree to which a clinical intervention or QI strategy was implemented as prescribed/intended [28]
	Perceptions of the solution and/or QI strategies	Perceptions among stakeholders that solutions and/or QI strategies were acceptable, appropriate, and/or feasible [28]
	Maintenance	Extent to which a newly implemented solution was sustained over time [33]
Effectiveness Outcomes	Service outcomes	Changes in the quality of services delivered, with quality encompassing efficiency, safety, effectiveness, equity, patient-centeredness, and timeliness [28]
	Resident outcomes	Changes in the health and wellbeing of NH residents [28]

### Synthesis of results

Data were entered into a matrix and organized so that publications reporting on a single study were grouped together. Studies then were organized by design: cluster randomized and controlled trials, non-randomized and controlled studies, and non-randomized and non-controlled studies. We used vote counting to identify the frequency that studies reported each type of QI strategy, implementation outcome, and statistically significant service and/or resident outcomes.

## Results

As indicated in the PRISMA-ScR diagram (Additional File 2), 77 articles on 59 studies met the inclusion criteria; characteristics of these 59 studies are presented in Table 2. Studies were conducted in the US (*n* = 41), Canada (*n* = 7), England (n = 4), and other countries (n = 7). The sample size ranged from 2 to 105 NHs, with a median of 12 NHs. Study designs included cluster randomized and controlled studies (*n* = 12), non-randomized and controlled studies (n = 12), and non-randomized and non-controlled studies (*n* = 35).

**Table 2:** Study Characteristics (*N* = 59 studies)

Papers	Setting	Problem	Project or Study Name	QI Strategies (count)	Implementation Outcomes (type)	Outcomes S = *p* < .05, NS = *p* ≥ .05, NR = S not reported	
						Service	Resident
Primary study design: Cluster randomized and controlled trial (includes between group differences in service and resident outcomes)							
Boyd, 2014 [61]	New Zealand, 29 NHs^a^	Hospital transfers	Residential Aged Care Integration Program	5	• Adoption • Reach to staff	none	Falls rate increased overall but less in Intvn.^b^ group **S**
Bravo, 2005 [62]	Canada, 40 NHs	Quality of care	none	6	• Adoption	Quality of care scores **NS**	none
Colon-Emeric, 2007 [63]	US, 67 NHs	Falls and fractures	none	4	• Adoption • Reach to residents	Use of hip protectors and pharmacotherapy **NS**	Falls rate **NS**
Colon-Emeric, 2013 [64]	US, 8 NHs	Falls	CONNECT for Quality	9	• Reach to staff • Reach to residents	• Communication and safety culture scores **S** • Falls risk reduction activities **NS**	none
Colon-Emeric, 2017 [65]; Colon-Emeric, 2016 [56]	US, 24 NHs	Falls	CONNECT for Quality	9	• Reach to staff and residents • Sustainment	Falls risk reduction activities **NS**	Falls rate **NS**
Crespy, 2016 [66]	US, 37 NHs	Symptoms of depression	Promoting Positive Well-Being	5	• Adoption • Reach to staff • Perceptions	none	Rate of symptoms of depression **S**
Kane, 2017 [67]; Huckfedlt, 2018 [68]; Tappen, 2018 [69]; Tappen, 2017 [70]	US,85 NHs	Hospital transfers	INTERACT	8	• Adoption • Reach to residents • Perceptions	Rate staff used tools for monitoring and communicating changes in health **NR**	• Hospital transfers **NS** • In NHs with high tool use, hospital transfers **S**
Kennedy, 2015 [71]; Kennedy, 2014 [72]	Canada, 12 NHs	Osteo-porosis and fractures	Vitamin D and Osteo-porosis Study	8	• Adoption • Reach to staff and residents	• Vitamin D and Calcium prescribing **S** • Osteoporosis medication prescribing **NS**	none
Nace, 2011 [73]	US, 6 NHs	Immuni-zation	none	5	• Reach to residents	Staff and resident immunization **NR**	none
Rantz, 2012 [74]; Rantz, 2012 [75]; Rantz; 2013 [76]	US, 29 NHs	Quality of care	none	8	• Adoption • Perceptions • Sustainment	Some subscales of the Observable Indicators of Quality scale, **S**	• Rate of Pressure ulcers, **S** • Other resident outcomes **NS**
Seers, 2018 [77]; Rycroft-Malone, 2018 [78]	England, Sweden, Nether-lands, Republic of Ireland, 24 NHs	Urinary incontinence	Facilitating Implemen-tation of Research Evidence	5	• Adoption • Reach to residents • Perceptions	Urinary continence treatment **S**	none
Tija, 2015 [79]	US, 42 NHs	Use of anti-psychotic medication	none	4	• Adoption • Reach to staff • Perceptions	Antipsychotic use **NS**	none
Primary study design: Non-randomized and controlled study (includes within group differences in services and resident outcomes)							
Arling, 2014 [80]; Abrahamson [81]	US, 15 NHs	Falls	none	5	• Perceptions	none	Falls rate **S**
Azermai, 2017 [82]	Belgium, 2 NHs	Psychotropic medications	Leiehome Project	2	• Reach to residents • Sustainment	• Rate of sedative use **S** • Rate of anti-psychotic use **NS**	none
Hanson, 2005 [83]	US, 9 NHs	End of life care	none	7	• Adoption • Reach to staff and residents	Rate of hospice enrollment, assessing/treating pain and discussions about end-of-life **S**	none
Jones, 2004 [84]	US, 12 NHs	Pain	none	5	• Adoption • Perceptions	The rate of pain assessments **S**	Rate of pain **NS**
Kaasalainen, 2012 [41]; Kaasalainen, 2015 [85]	Canada, 4 NHs	Pain	none	6	• Reach to residents • Perceptions	Rate of pain assessment tool use and initial pain assessments **S**	Rate of pain increased overall but less in Intvn. group **S**
Olsho, 2014 [86]	US, 25 NHs	Pressure ulcers	none	7	• Adoption • Reach to residents	none	Rate of pressure ulcers **S**
Rantz, 2018 [35]; Rantz, 2017 [87]; Flesner, 2019 [88]; Popejoy, 2017 [89]; Vogelsmeier, 2015 [90]	US, 16 NHs	Hospital transfers	Missouri Quality Initiative	9	• Reach to residents • Perceptions	Rate of antipsychotic use **S**	• Composite indicator of resident outcomes **S** • Rate of hospital transfers **S**
Rask, 2007 [91]	US, 42 NHs	Falls	none	7	• Reach to residents	Rate of documented falls risk assessment and management **S**	Falls rate was unchanged in Intvn group but increased in control group **S**
Sales, 2014 [92]; Sales, 2015 [93]	Canada, 4 NHs	Falls	none	4	• Reach to residents • Perceptions	none	Falls rate increased **S**
Sheaff, 2018 [38]	England, 23 NHs	Dementia care	Dementia Learning Community	5	• Reach to staff and residents • Perceptions	Staff knowledge of dementia **NS**	Quality of life of residents with dementia **NS**
Unroe, 2018 [36]	US, 40 NHs	Hospital transfers	OPTIMISTIC	8	• Adoption • Reach to staff	Use of new billing codes for medical care of varied **NR**	none
Zimmerman, 2014 [37]	US, 12 NHs	Antibiotic use	none	7	• Reach to staff and residents	Rate of prescribing antibiotics decreased overall all but more the Intvn group **S**	none
Primary study design: Non-randomized and non-controlled study							
Abel, 2005 [94]	US, 20 NHs	Pressure ulcers	none	5	• Adoption • Reach to residents • Perceptions	Rate of pressure ulcer prevention and treatment **S**	Rate of pressure ulcers **NS**
Badger, 2009 [95]	England, 49 NHs	End of life care	Gold Standards Framework in Care Homes programme	4	• Adoption • Reach to residents • Perceptions	Rate of advance care planning **S**	Rate of NH as the place of death **S**
Baier, 2003 [96]	US, 29 NHs	Pressure ulcers	Northeast Pressure Ulcer Project	8	• Adoption • Reach to residents • Perceptions	Rate of pressure ulcer prevention and treatment **S**	none
Baier, 2004 [97]	US, 17 NHs	Pain	none	7	• Adoption • Reach to residents • Perceptions	• Rate of pain assessment and non-pharmacologic pain treatment **S** • Rate of pharmacologic pain treatment **NS**	Prevalence of pain **S**
Boyle, 2013 [98]	US, 2 NHs	Diabetes care	none	3	• Reach to residents • Perceptions	Rate of diabetes care **S**	Rate of hypoglycemia **S**
Bravo, 2005 [99]	Canada, 18 NHs	Quality of care	none	5	• Perceptions	Goal Attainment scores **NS**	none
Buhr, 2006 [100]	US, 4 NHs	Pain	none	6	• Reach to staff	Rate of pain assessments **NR**	Patient satisfaction with pain care **NS**
Carson, 2017 [101]	Canada, 7 NHs	Emergent transfers	London Transfer Project	7	• Perceptions	Rate of documenting rationale of emergency department transfers **NR**	none
Chodash, 2015 [102]	US, 5 NHs	Depression	Practice Improve-ment Education Project	6	• Adoption • Reach to staff and residents	• Knowledge of depression care **S** • Rate of anti-depressant use **S**	
Colon-Emeric, 2006 [103]	US, 36 NHs	Falls	none	6	• Adoption • Reach to residents	• Rate of assessing fall risk and prescribing Vitamin D **S** • Rate of fall risk reduction strategies and prescribing Calcium **NS**	Falls rate **NS**
Dolansky, 2013 [104]	US, 4 NHs	Heart failure	none	6	• Reach to residents • Perceptions	Rate of heart failure treatment in one but not all NHs **NR**	none
Edwards, 2017 [105]	Australia, 7 NHs	Wound and skin care	Champions for Skin Integrity	5	• Perceptions	Rate of wound management and prevention strategies **S**	The rate of wounds **S**
Fallon, 2006 [106]	Australia, 2 NHs	Oral health	none	6	• Reach to staff • Perceptions	Staff knowledge of 2 but not all oral health care procedures **S**	none
Fine, 2014 [107]	US 8 NHs	Pain	none	5	• Reach to staff and residents • Perceptions	• Care plan with pain goals, **S** • Other processes, **NS**	none
Fitzler, 2016 [39]	US, 30 NHs	Dementia care	none	6	• Perceptions	Scores on 9 of 10 quality indicators **S**	Rate of ‘resident-on-staff’ altercations **S**
Hartmann, 2018 [40]	US, 6 NHs	Person-centered care	LOCK model	6	• Reach to residents • Perceptions	Number of opportunities for staff and resident interactions **S**	Rate of negative staff and resident interactions **NS**
Hickman, 2016 [108]; Ersek, 2018 [109]	US, 19 NHs	Advance care planning	OPTIMISTIC	8	• Reach to staff and residents • Perceptions	Rate of advance care planning and changes in written advance care plans **NR**	none
Horn, 2010 [110]; Sharkey, 2013 [111]	US, 11 NHs	Pressure ulcers	Real-Time	7	• Reach to residents • Fidelity • Perceptions	Time that certified nursing assistants stayed late to complete documentation **S**	Rate of high risk residents with pressure ulcers and the rate of new pressure ulcers **S**
Horner, 2005 [112]	US, 9 NHs	Pain	none	3	• Adoption	• Rate of pain assessment and non-pharmacologic pain treatment **S** • Rate of pharmacologic pain treatment **NS**	none
Keeney, 2008 [113]	US, 4 NHs	Pain	none	6	• Sustainment	• Rate of pain assessment **S** • Rate of pain treatment **NS**	none
Kezerian, 2018 [114]	Canada, 15 NHs	Hospital transfers	Palliative Care in Residential Care Initiative	5	• Reach to staff • Perceptions	Rate of 1 of 5 advance care planning services **S**	Rate of hospital transfers **NS**
Kovach, 2008 [115]	US, 8 NHs	Pain	Serial Trial Intervention	3	• Reach to staff • Perceptions • Sustainment	Rate of pain assessment and treatment **S**	Rate of behavioral symptoms **NR**
Lai, 2018 [116]	Taiwan, 11 NHs	Hand hygiene	none	6	• Reach to residents • Fidelity	none	The patient infection density **S**
Lynn, 2007 [117]	US, 35 NHs	Pressure ulcers		8	• Adoption	Rate of pressure ulcer risk assessment **S**	• Rate of Stage III and IV pressure ulcers S • Rate of Stage I and II pressure ulcers **NS**
Ouslander, 2009 [118]	US, 3 NHs	Hospital transfers	INTERACT II	7	• Perceptions	none	Rate of hospital transfers **NR**
Rask, 2017 [91]	US, 105 NHs	Hospital transfers	INTERACT II	5	• Perceptions	none	Hospital transfers in 2 of 3 organizations **S**
Rosen, 2005 [119]; Rosen, 2006 [120]	US, 2 NHs	Pressure ulcers	none	6	• Reach to staff	none	• Rate of pressure ulcers (Stage II or greater) **S** • Rate of pressure ulcers equivalent for Black and White residents, **S**
Sand, 2007	US, 15 NHs	Staff immunization	none	5	• Adoption • Perceptions	Rate of immunization, **NS**	none
Scott-Cawiezell, 2009 [121]	US, 5 NHs	Nursing care	none	5	• Reach to residents • Perceptions	Monitoring implementation of an electronic medical records system **NR**	none
Simmons, 2013 [122]	US, 2 NHs	Weight loss	none	9	• Reach to staff and residents • Perceptions	Quantity of food consumed and the rate staff offered alternative meal choices **S**	Daily weight loss **NS**
Tena-Nelson, 2012 [123]	US, 18 NHs	Hospital transfers	INTERACT	5	• Adoption • Reach to staff and residents • Perceptions	Rate staff used tools for monitoring and communicating changes in health **NR**	Rate of hospital transfers **NS**
Torma, 2014 [124]	Sweden, 4 NHs	Nutrition	none	6	• NR	none	Nutritional status scores **NS**
Triller, 2014 [125]	US, 12 NHs	Anti-coagulant therapy	none	7	• Adoption • Perceptions	Rate of timely post-antibiotic prescribing international normalised testing **S**	none
Wilson, 2018 [126]	England 2 NHs	Hydration	none	3	• Perceptions	Rate fluids offered **NR**	Rate of adverse health events **NR**
Zubkoff, 2018 [127]	US, 21 NHs	Falls	none	7	• Perceptions	Rate of “post-fall’ huddles **NR**	Falls rate **NS**

Key: ^a^ nursing home, ^b^ intervention

### Clinical problems and solutions

Studies of QI focused on 23 clinical problems in care of frail, older adults; most commonly, pressure ulcers (*n* = 8), falls (n = 8), pain (n = 8), and hospital transfers (*n* = 7). Solutions to address these problems were enacted by NH staff working on inter-disciplinary teams. In 56 studies (95%), team members included existing NH staff, such as physicians, nursing assistants, nurses and nurse practitioners, pharmacists, and social workers. In three studies, nurses and/or nurse practitioners were added to existing NH teams to deliver new care practices and support the work of others. In 16 studies (27%), the solution was a practice guideline or intervention protocol, such as the Falls Management Program [34]. In other studies (73%), the solution was reported as a synthesis of evidence from practice guidelines, systematic reviews, clinical trials, and/or pilot studies. Moreover, in some studies solutions included a synthesis of evidence and added staff members, for example, OPTIMISTIC and the Missouri Quality Initiative [35, 36]. Across studies, reports on the characteristics of solutions varied widely and often did not identify an intervention protocol for improving care.

### QI strategies

**Fig. 2:**
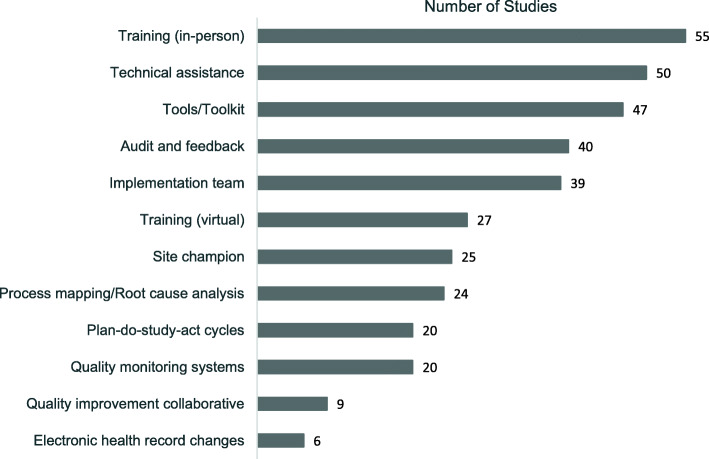
Frequency of Quality Improvement Strategies (*N* = 59 Studies)

Studies included a range of QI strategies to support uptake or sustainment of clinical solutions (Fig. 2); an average of 6 to 7 QI strategies were used in each study. The most frequently reported strategies were in-person training (*n* = 55), technical assistance (*n* = 50), tools/toolkits (*n* = 47), audit and feedback (*n* = 40), and implementation teams (*n* = 39). In 42 studies (71%), authors reported using a bundle of three QI strategies that included tools/toolkits, in-person training, and technical assistance. In contrast, other QI strategies were reported less frequently; PDSA cycles were reported in 20 studies (34%) and modifications in electronic health records systems were reported in 6 studies (10%).

### Implementation outcomes

**Fig. 3:**
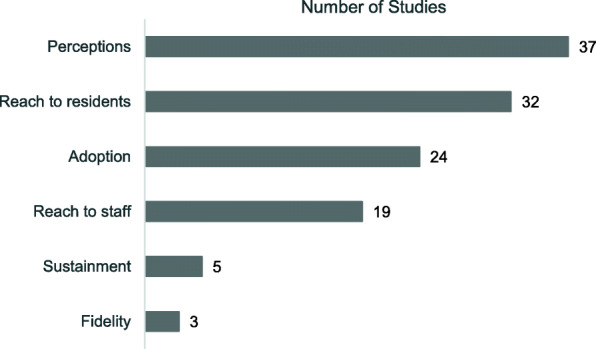
Frequency of Implementation Outcomes (N = 59 Studies)

Fifty-eight studies (98%) included descriptions of implementation outcomes (Fig. 3), and an average of two implementation outcomes was reported per study. The most frequently reported outcome was NH staff perceptions of the feasibility, acceptability, or satisfaction with the clinical intervention and/or the QI strategies (*n* = 37). Other more common implementation outcomes were reach to residents (*n* = 32), setting adoption (*n* = 24), and reach to staff [20]. Comparison of these outcomes across studies was limited by variability in how outcomes were measured. For example, a common pattern of reporting reach to staff and reach to residents was the number of staff trained or residents who received new services, as opposed to the rate that eligible staff were trained or eligible residents received new services. Finally, the outcome, fidelity to intervention protocols, was rarely reported.

### Service and resident outcomes

Articles from 49 of 59 studies (85%) included descriptions of service outcomes, such as improving the quality of falls prevention or pain prevention and management services. Of the 49 studies reporting service outcomes, 37 studies included tests of statistical significance of change; 31 of these 37 studies (84%) indicated significant improvements in at least one service outcome. Across these 31 studies, 4 studies used randomized and controlled designs and 27 studies (87%) used non-randomized and controlled designs or non-randomized and non-controlled designs. More commonly reported improvements in service outcomes were the quality of services related to pain (*N* = 7), pressure ulcers (*N* = 3), advance care planning or end-of-life care (N = 3), and changes in medication prescribing (*N* = 4), such as antibiotic or antipsychotic medication. Moreover, articles from 34 of 59 studies included descriptions of resident outcomes (e.g., falls rate and rate of pressure ulcers). Of these, 33 of 34 studies included tests of statistical significance; 20 of 33 studies (61%) indicated significant improvement in at least one resident outcome. Among the 20 studies demonstrating significant improvement, the more commonly improved resident outcomes were pressure ulcers (*N* = 5), hospital transfers (*N* = 3), and resident falls (*N* = 2).

## Discussion

In this scoping review of peer-reviewed articles of QI in NHs, we identified patterns in the types of quality problems addressed in NHs, solutions selected to target those problems, QI strategies used to implement solutions, and the impact that solutions and QI strategies had on implementation, service, and client outcomes. As discussed below, several features of the literature and our review methods limited our ability to fully map how QI strategies are being used in NH. Despite these limitations, the review provides a foundation for understanding how QI strategies are used and suggests practical steps to improve future QI and implementation studies in NHs.

### Limitations

The potential for publication bias was a major limitation in this review. A large majority of QI work in NHs is not meant for publication and is not reflected in this review, which was limited to peer-reviewed articles reporting on QI studies. Moreover, many published reports likely had external funding and may not be generalizable to QI across NHs. Another limitation is that terminology is inconsistently applied in the QI literature and this limits efforts to extract data and synthesize findings across studies. In this review, we opted to be broadly inclusive in both our study selection and data extraction. As result, we included a diversity of studies, including studies of intervention effectiveness that a more conservative definition of quality improvement studies might have excluded. This fit the goal of the scoping review, which was to map how QI methods are being used in published research in NHs. In extracting data, we were particularly liberal in our classification of implementation outcomes. For example, when studies reported the number of NH residents that received new services, we classified this as the implementation outcome “reach,” even when authors did not identify it as an outcome or did provide other elements of reach, such as the reporting on the proportion of eligible residents who received the service. Furthermore, we encountered challenges in our use of the Framework for Implementation Research to categorize attributes of QI reports. While some aspects of QI and implementation science overlap, the distinction between “what” investigators choose to implement (solutions/interventions) and “how” they implement it” (implementation strategies) is not always a characteristic of QI. Authors frequently integrated reports of clinical solutions and QI strategies which made is difficult to extract the two as separate phenomenon. Further, authors often presented evidence of multiple service and/or resident outcomes; we coded outcomes as effective if evidence that at least one outcome indicated improvement; thus, our findings may over-state study outcomes. Several strengths of our study procedures reduced the occurrence and the impact of these risks of error; for example, we used an evidence-informed codebook to categorize solutions, QI strategies, implementation, service, and resident outcomes. Further, two investigators independently coded all reports and disagreements in coding decisions were resolved in discussion. Finally, we used a team process to generate and describe patterns in the synthesis of study findings; this included reviews of data in our data matrix, study tables and figures, and the narrative report of study findings.

A summary of review findings and the fit of findings with prior research are described below.

### Problems addressed

The 59 studies addressed a range of care problems in NHs, with pressure ulcers, falls, pain, and hospital transfers among the problems most frequently addressed. Many enduring NH care problems were under-studied, such as antibiotic stewardship [37] and support for people living with dementia [38–40]. Similarly, our study did not capture any studies on the topic of isolation and only one study of quality of life, suggesting opportunities for future improvement programs.

### Solutions selected to target problems

Most articles included few details about the solution and solutions were reported as a synthesis of evidence from multiple sources. Indeed, only 27% of studies examined improvement with specific interventions or practice guidelines; for example, in a QI program to improve pain management, Kaasalainen et al. reported the use of a protocol based on clinical practice guidelines published by the American Medical Directors Association and the American Geriatrics Society [41]. The lack of information on solutions limits the ability of others to replicate or compare solutions across studies. One explanation is that QI historically has focused on generating local solutions that are not intended to be generalized [17, 23]. As such, the intent of many QI reports is to share the process used to arrive at the solution rather than the solutions themselves. This was reflected in our finding that descriptions of clinical solutions and QI strategies frequently were reported together.

### QI strategies used to implement solutions

Authors described using an average of 6–7 QI strategies to implement solutions and address clinical problems. Authors were more likely to describe the strategies used by research teams and others external to the NH (e.g., tools, training, and technical assistance) than they were to describe the strategies used by staff internal to the NH (e.g., implementation teams, process mapping, root cause analysis, and PDSA cycles). With the exception of implementation teams, our findings indicate that internal NH strategies were used in less than half the studies. These findings are consistent with earlier research in NHs [19–21] and prior reviews on the limited use of PDSA cycles in QI studies in other settings [26, 42].

The disproportionate focus on QI strategies used by those external to NHs, as compared to those used by staff in NHs, may be an area for improvement. QI studies are time-limited and, at the end of the study, those providing training, technical assistance, and other externally delivered strategies often move on to the next study. For changes in NHs to be sustained over time, NH staff must be able to engage in QI strategies and continue monitoring a problem and its solution and overcoming barriers over time [43]. Greater attention to NH internal strategies also has the potential to build capacity of NH staff to apply QI when new problems arise [44]. Describing internal QI strategies also is critical to understanding the causal pathway through which external QI strategies affect change in service and client outcomes [45]. For example, to what extent do NH staff who participate in a QI collaborative complete the recommended internal QI strategies (e.g., conduct PDSA cycles to iteratively develop and test potential solutions)? Among reviewed studies, Hartmann and colleagues exemplify the value of studying both external and internal QI strategies. The study team trained NH staff to conduct QI cycles using the “LOCK” model (Look for bright spots, Observations by everyone, Collaborate in huddles, and Keep it bite sized) [46]. The study team also evaluated staff use of the LOCK model. Findings indicated this approach helped staff appraise the advantages of new care practices and learn how to apply them with NH residents [40].

### Implementation outcomes

On average, studies reported findings on two implementation outcomes, with 63% of studies reporting on NH staff perceptions of participating in QI programs or using new solutions, 54% of studies reporting on the reach of new care practices (solutions) to residents, 41% reporting on NH adoption, and 3% reported on fidelity to written protocols. These findings accord with evidence in reviews of QI studies in other settings [15, 21]; for example, fidelity was described in fewer than half of reports on randomized trials of QI initiatives to improve management of chronic kidney disease [47].

Evaluating the impact of QI strategies on implementation outcomes is necessary to answer questions about when and how QI strategies work in NHs [17, 28, 48]. For example, how many and what types of NH staff must be reached for QI strategies to improve service and resident outcomes? What type and dose (e.g., duration and frequency) of QI strategies increase the proportion of eligible residents reached by a clinical solution and promote equitable reach across subpopulations? In this review, exemplars of the practical utility of measuring implementation outcomes included a study of Zimmerman and colleagues, who reported a successful QI program in 6 NHs to reduce antibiotic prescribing [37]. The outcomes of this program were in part attributed to the wide reach of antibiotic stewardship training, which reached more than half of the physicians and nurses providing care in the NHs. Consistent with prior literature [18], rigorous measurement of implementation outcomes provided essential data to explain the impact of QI strategies on service and resident outcomes.

### Service and resident outcomes

A major challenge for studying QI is that the observational design of most studies may not account for factors outside of investigator control that influence the impact of solutions on outcomes; moreover, few are designed with sufficient power to avoid a type I or type II error [49]. Thus, findings in this review, which suggest that half of QI studies significantly improved service or resident outcomes, likely include substantial risk of bias. These findings support earlier research in NHs [20, 21]. However, the findings should be interpreted cautiously, recognizing that QI is usually focused on incremental changes to overcome local problems, and not statistical power. An additional limitation in studies was the tendency to compare outcomes before and after the start of the QI program, when analysis of change over time, using run charts and other longitudinal approaches, may provide more accurate data about performance [17].

### Recommendations for future research and practice

Review findings suggest several implications for future research and practice. First, reporting of results would be improved by following the SQUIRE or other guidelines for reporting QI studies [48, 50]. Of note, the SQUIRE guidelines define “interventions” broadly to include both clinical interventions and QI interventions (i.e., QI strategies). To avoid confusion, we recommend that authors clearly distinguish between clinical and QI intervention activities and provide a summary of the evidence in support of their clinical interventions, including citations to prior relevant studies. Efforts to replicate and synthesize the findings from QI studies also may benefit from recent advances in implementation science. Guidelines for reporting implementation strategies could also be applied to QI strategies, including recommendations to report the actor (who enacted the strategy), action (specific activities involved), and action target (the specific barrier or facilitator that the action is intended to change) [51]. In reporting QI strategies, we further recommend that authors distinguish between the strategies enacted by intermediaries external to the NH and those enacted by staff internal to the NH [52]. Lastly, we recommend that authors of QI studies consider using existing taxonomies of implementation outcomes to improve consistency in how they are named, defined, and operationalized [28, 33].

In addition to recommendations for improving the reporting of QI studies, our findings suggest several opportunities for future research. First, NHs are required to develop Quality Assurance Programs Improvement programs (QAPI); yet, little is known about the extent to which NHs have developed QAPI infrastructure or how it varies. Research is needed to understand how QAPI in NHs is functioning so that QI initiatives can be designed to align with, build, and leverage existing QI capacity; for example, evidence in a national QAPI registry could be used to describe and evaluate of QAPI programs. Second, studies in this review used multiple QI strategies and those strategies were enacted by both NH staff and intermediary organizations. Multi-level research studies are needed to understand how these strategies interact and to identify which bundles of strategies are most effective under what circumstances [53]. Moreover, future systematic reviews may be needed to describe multi-level strategies and improvement related to specific problems, such as falls, pain, and hospital transfers. Third, if evidence-based practitioners are going to spread findings from QI studies, there must be a way to measure and report how the QI was implemented even though that is not a typical part of the methodology. For example, new approaches for evaluating and reporting fidelity and adaption are needed to identify whether clinical interventions and QI strategies were delivered as intended as well as how and why they were adapted. This information is key to understanding how clinical interventions and QI strategies work and to identify opportunities for further refinement [54, 55]. Fourth, as noted in previous research [44, 56], future studies are needed that assess the sustainment of improvement over time. Studies also are needed to characterize the context of care in NHs and describe contextual factors that interact with QI programs and influence outcomes [57], for example, NH administration, organizational structure, health records systems, and coordination with medical staff. Finally, future reviews of QI in NHs are needed to describe (1) QI programs that are not in peer-reviewed publications (2), involvement of family caregivers in QI (3), sources of funding and author affiliations for published studies, and (4) the extent to which SQUIRE guidelines are followed.

## Conclusions

The purpose this review was to map-out QI research in NHs and to offer preliminary guidance for future studies designed to promote the replication and synthesis of promising solutions. This review also provides recommendations for refining procedures for more effective improvement work in NHs. While the reports of QI in NHs and elements of this review had limitations, QI was observed as a promising approach to improve care for older adults in NHs.

## Supplementary Information

**Additional file 1.**

**Additional file 2.**

## Data Availability

The datasets used and/or analyzed in the current study are available from the corresponding author on reasonable request.

## Abbreviations

NC: North Carolina
QI: Quality improvement
NH: Nursing homes
PDSA: Plan-do-study-act
US: United States
PRISMA-ScR: Preferred Reporting Items for Systematic Reviews and Meta-Analysis extension for Scoping Reviews
CINAHL: Cumulative Index to Nursing and Allied Health Literature
EBSCO: Elton B. Stephens Company
QAPI: Quality Assurance and Performance Improvement

## References

[1] Harris-Kojetin L SM, Lendon JP, Rome V, Valverde R, Caffrey C. Long-term care providers and services users in the United States, 2015–2016 2019 [Available from: https://www.cdc.gov/nchs/data/series/sr_03/sr03_43-508.pdf]. 27023287

[2] National Institute on Aging. Long-term care, residential facilities, assisted living, and nursing homes, 2017 [Available from: https://www.nia.nih.gov/health/residential-facilities-assisted-living-and-nursing-homes].

[3] Zarowitz BJ, Resnick B, Ouslander JG (2018). Quality clinical Care in Nursing Facilities. J Am Med Dir Assoc.

[4] Fashaw SA, Thomas KS, McCreedy E, Mor V. Thirty-year trends in nursing home composition and quality since the passage of the Omnibus Reconciliation Act. J Am Med Dir Assoc. 2020;21(2):233–9]. 10.1016/j.jamda.2019.07.004 PMC6986999 31451383

[5] Institute of M. Improving the quality of care in nursing homes. Washington, DC: The National Academies Press; 1986. 25032432

[6] Parmelee PA (2004). Quality improvement in nursing homes: the elephants in the room. J Am Geriatr Soc.

[7] Werner RM, Konetzka RT (2010). Advancing nursing home quality through quality improvement itself. Health Aff (Millwood).

[8] Harrington C (2017). Wiener.

[9] Research Triangle Institute. International. Nursing home care quality: twenty years after the Omnibus Budget Reconciliation Act of 1987 2007 [Available from: https://www.kff.org/wp-content/uploads/2013/01/7717.pdf].

[10] Park J, Konetzka RT, Werner RM (2011). Performing well on nursing home report cards: does it pay off?. Health Serv Res.

[11] Weech-Maldonado R, Pradhan R, Dayama N, Lord J, Gupta S (2019). Nursing home quality and financial performance: is there a business case for quality?. Inquiry..

[12] Konetzka RT, Davila H, Brauner DJ, Cursio JF, Sharma H, Werner RM (2021). The quality measures domain in nursing home compare: is high performance meaningful or misleading? Gerontologist.

[13] Lee JT, Polsky D, Fitzsimmons R, Werner RM (2020). Proportion of racial minority patients and patients with low socioeconomic status cared for by physician groups after joining accountable care organizations. JAMA Netw Open.

[14] Liao JM, Navathe AS, Werner RM (2020). The impact of medicare's alternative payment models on the value of care. Annu Rev Public Health.

[15] Alexander JA, Hearld LR (2009). What can we learn from quality improvement research? A critical review of research methods. Med Care Res Rev.

[16] Centers for Medicare and Medicaid Services. QAPI Five Elements 2016 [Available from: https://www.cms.gov/Medicare/Provider-Enrollment-and-Certification/QAPI/qapidefinition].

[17] Langley GJ, Moen, R.D., Nola, K.M., Nolan, T.W., Norman, C.L., Provost, L.P. The improvement guide: a practical approach to enhancing organizational performance. 2nd ed. San Francisco: Jossey-Bass; 2009.

[18] Carpenter CR, Pinnock H (2017). StaRI aims to overcome knowledge translation inertia: the standards for reporting implementation studies guidelines. J Am Geriatr Soc.

[19] Aylward S, Stolee P, Keat N, Johncox V (2003). Effectiveness of continuing education in long-term care: a literature review. Gerontologist..

[20] Caspar S, Cooke HA, Phinney A, Ratner PA. Practice change interventions in long-term care facilities: What works, and why? Can J Aging = La revue canadienne du vieillissement. 2016;35(3):372–84. 10.1017/S0714980816000374 27452374

[21] Low LF, Fletcher J, Goodenough B, Jeon YH, Etherton-Beer C, MacAndrew M (2015). A systematic review of interventions to change staff care practices in order to improve resident outcomes in nursing homes. PLoS One.

[22] Proctor EK, Landsverk J, Aarons G, Chambers D, Glisson C, Mittman B (2009). Implementation research in mental health services: an emerging science with conceptual, methodological, and training challenges. Admin Pol Ment Health.

[23] Koczwara B, Stover AM, Davies L, Davis MM, Fleisher L, Ramanadhan S (2018). Harnessing the synergy between improvement science and implementation science in cancer: a call to action. J Oncol Pract.

[24] Nilsen P BM, Thor J, Leeman J, Andersson G, Sevdalis N. Improvement science versus implementation science. In: Birken P, editor. Handbook on implementation science. UK: Edward Elgar Publishing; 2020.

[25] Nilsen P, Bender M, Thor J, Leeman J, Andersson G, Sevdalis N, Nilsen P, Birken S (2020). Improvement science versus implementation science. Handbook of implementation science.

[26] Taylor MJ, McNicholas C, Nicolay C, Darzi A, Bell D, Reed JE (2014). Systematic review of the application of the plan-do-study-act method to improve quality in healthcare. BMJ Qual Saf.

[27] Lewis CC, Proktor, E.K., Brownson, R.C. Measurement issues in dissemination and implementation research. In: Brownson RC, Colditz, G.A. Proctor, E.K., editor. Dissemination and implementation research in health: translating science to practice. NY: Oxford University Press; 2017.

[28] Proctor E, Silmere H, Raghavan R, Hovmand P, Aarons G, Bunger A (2011). Outcomes for implementation research: conceptual distinctions, measurement challenges, and research agenda. Admin Pol Ment Health.

[29] Tricco AC, Lillie E, Zarin W, O'Brien KK, Colquhoun H, Levac D (2018). PRISMA extension for scoping reviews (PRISMA-ScR): checklist and explanation. Ann Intern Med.

[30] Veritas Health Innovation. Covidence systematic review software. Melbourne, Australia.

[31] Powell BJ, Waltz TJ, Chinman MJ, Damschroder LJ, Smith JL, Matthieu MM (2015). A refined compilation of implementation strategies: results from the expert recommendations for implementing change (ERIC) project. Implement Sci.

[32] Wandersman A, Chien VH, Katz J (2012). Toward an evidence-based system for innovation support for implementing innovations with quality: tools, training, technical assistance, and quality assurance/quality improvement. Am J Community Psychol.

[33] Kessler RS, Purcell EP, Glasgow RE, Klesges LM, Benkeser RM, Peek CJ (2013). What does it mean to "employ" the RE-AIM model?. Eval Health Prof.

[34] Taylor JA, Parmelee P, Brown H, Strothers HS, Capezuti E, Ouslander JG (2007). A model quality improvement program for the management of falls in nursing homes. J Am Med Dir Assoc.

[35] Rantz MJ, Popejoy L, Vogelsmeier A, Galambos C, Alexander G, Flesner M (2018). Impact of advanced practice registered nurses on quality measures: the Missouri quality initiative experience. J Am Med Dir Assoc.

[36] Unroe KT, Fowler NR, Carnahan JL, Holtz LR, Hickman SE, Effler S (2018). Improving nursing facility care through an innovative payment demonstration project: optimizing patient transfers, impacting medical quality, and improving symptoms: transforming institutional care phase 2. J Am Geriatr Soc.

[37] Zimmerman S, Sloane PD, Bertrand R, Olsho LE, Beeber A, Kistler C (2014). Successfully reducing antibiotic prescribing in nursing homes. J Am Geriatr Soc.

[38] Sheaff R, Sherriff I, Hennessy CH (2018). Evaluating a dementia learning community: exploratory study and research implications. BMC Health Serv Res.

[39] Fitzler S, Raia P, Buckley FO, Wang M (2016). Does nursing facility use of habilitation therapy improve performance on quality measures?. Am J Alzheimers Dis Other Dement.

[40] Hartmann CW, Mills WL, Pimentel CB, Palmer JA, Allen RS, Zhao S (2018). Impact of intervention to improve nursing home resident-staff interactions and engagement. Gerontologist..

[41] Kaasalainen S, Brazil K, Akhtar-Danesh N, Coker E, Ploeg J, Donald F, et al. The evaluation of an interdisciplinary pain protocol in long lerm care. J Am Med Dir Assoc. 2012;13(7):664.e1–8. 10.1016/j.jamda.2012.05.013 22739020

[42] Knudsen SV, Laursen HVB, Johnsen SP, Bartels PD, Ehlers LH, Mainz J (2019). Can quality improvement improve the quality of care? A systematic review of reported effects and methodological rigor in plan-do-study-act projects. BMC Health Serv Res.

[43] Chambers DA, Glasgow RE, Stange KC (2013). The dynamic sustainability framework: addressing the paradox of sustainment amid ongoing change. Implement Sci.

[44] Bakerjian D, Zisberg A (2013). Applying the advancing excellence in America's nursing homes circle of success to improving and sustaining quality. Geriatr Nurs.

[45] Leeman J, Toles M (2020). What does it take to scale-up a complex intervention? Lessons learned from the Connect-home transitional care intervention. J Adv Nurs.

[46] Mills WL, Pimentel CB, Palmer JA, Snow AL, Wewiorski NJ, Allen RS (2018). Applying a theory-driven framework to guide quality improvement efforts in nursing homes: the LOCK model. Gerontologist..

[47] Silver SA, Bell CM, Chertow GM, Shah PS, Shojania K, Wald R (2017). Effectiveness of quality improvement strategies for the management of CKD: a meta-analysis. Clin J Am Soc Nephrol.

[48] Ogrinc G, Davies L, Goodman D, Batalden P, Davidoff F, Stevens D (2016). SQUIRE 2.0 (standards for QUality improvement reporting excellence): revised publication guidelines from a detailed consensus process. BMJ Qual Saf.

[49] Guyatt G, Rennie D, Meade MO, Cook DJ (2015). Contributors. Users' guides to the medical literature: a manual for evidence-based clinical practice, 3rd ed.

[50] Pinnock H, Barwick M, Carpenter CR, Eldridge S, Grandes G, Griffiths CJ (2017). Standards for reporting implementation studies (StaRI) statement. BMJ..

[51] Proctor EK, Powell BJ, McMillen JC (2013). Implementation strategies: recommendations for specifying and reporting. Implement Sci.

[52] Leeman J, Birken SA, Powell BJ, Rohweder C, Shea CM (2017). Beyond "implementation strategies": classifying the full range of strategies used in implementation science and practice. Implement Sci.

[53] Powell BJ, Fernandez ME, Williams NJ, Aarons GA, Beidas RS, Lewis CC (2019). Enhancing the impact of implementation strategies in healthcare: a research agenda. Front Public Health.

[54] Leviton LC, Trujillo MD (2017). Interaction of theory and practice to assess external validity. Eval Rev.

[55] Stirman SW, Miller CJ, Toder K, Calloway A (2013). Development of a framework and coding system for modifications and adaptations of evidence-based interventions. Implementation Sci..

[56] Colon-Emeric C, Toles M, Cary MP, Batchelor-Murphy M, Yap T, Song Y (2016). Sustaining complex interventions in long-term care: a qualitative study of direct care staff and managers. Implementation sci..

[57] Damschroder LJ, Aron DC, Keith RE, Kirsh SR, Alexander JA, Lowery JC (2009). Fostering implementation of health services research findings into practice: a consolidated framework for advancing implementation science. Implementation Sci.

[58] Brown CH, Curran G, Palinkas LA, Aarons GA, Wells KB, Jones L (2017). An overview of research and evaluation designs for dissemination and implementation. Annu Rev Public Health.

[59] Leeman J, Calancie L, Hartman MA, Escoffery CT, Herrmann AK, Tague LE (2015). What strategies are used to build practitioners' capacity to implement community-based interventions and are they effective?: a systematic review. Implement Sci.

[60] Institute for Healthcare Improvement. Flowchart 2020 [Available from: http://www.ihi.org/resources/Pages/Tools/Flowchart.aspx].

[61] Boyd M, Armstrong D, Parker J, Pilcher C, Zhou L, McKenzie-Green B (2014). Do gerontology nurse specialists make a difference in hospitalization of long-term care residents? Results of a randomized comparison trial. J Am Geriatr Soc.

[62] Bravo G, Dubois M-F, Roy P-M (2005). Using goal attainment scaling to improve the quality of long-term care: a group-randomized trial. Int J Qual Health Care.

[63] Colon-Emeric CS, Lyles KW, Levine DA, Schenck AP, Allison J, House P (2007). Randomized trial to improve fracture prevention in nursing home residents. Am J Med.

[64] Colon-Emeric CS, McConnell E, Pinheiro SO, Corazzini K, Porter K, Earp KM (2013). CONNECT for better fall prevention in nursing homes: results from a pilot intervention study. J Am Geriatr Soc.

[65] Colon-Emeric CS, Corazzini K, McConnell ES, Pan W, Toles M, Hall R, et al. Effect of promoting high-quality staff interactions on fall prevention in nursing homes: A cluster-randomized trial 2017;177(11):1634–1641. 10.1001/jamainternmed.2017.5073 PMC5710274 28973516

[66] Crespy SD, Van Haitsma K, Kleban M, Hann CJ (2016). Reducing depressive symptoms in nursing home residents: evaluation of the Pennsylvania depression collaborative quality improvement program. J Healthc Qual.

[67] Kane RL, Huckfeldt P, Tappen R, Engstrom G, Rojido C, Newman D (2017). Effects of an intervention to reduce hospitalizations from nursing homes: a randomized implementation trial of the INTERACT program. JAMA Intern Med.

[68] Huckfeldt PJ, Kane RL, Yang Z, Engstrom G, Tappen R, Rojido C, et al. Degree of implementation of the interventions to reduce acute care transfers (INTERACT): Quality improvement program associated with number of hospitalizations. 2018;66(9):1830–7. 10.1111/jgs.15476 PMC6156928 30094818

[69] Tappen RM, Newman D, Huckfeldt P, Yang Z, Engstrom G, Wolf DG (2018). Evaluation of nursing facility resident safety during implementation of the INTERACT quality improvement program. J Am Med Dir Assoc.

[70] Tappen RM, Wolf DG, Rahemi Z, Engstrom G, Rojido C, Shutes JM (2017). Barriers and facilitators to implementing a change initiative in long-term care using the INTERACT quality improvement program. Health Care Manag.

[71] Kennedy CC, Ioannidis G, Thabane L, Adachi JD, Marr S, Giangregorio LM (2015). Successful knowledge translation intervention in long-term care: final results from the vitamin D and osteoporosis study (ViDOS) pilot cluster randomized controlled trial. Trials..

[72] Kennedy CC, Thabane L, Ioannidis G, Adachi JD, Papaioannou A (2014). Implementing a knowledge translation intervention in long-term care: feasibility results from the vitamin D and osteoporosis study (ViDOS). J Am Med Dir Assoc.

[73] Nace DA, Perera S, Handler SM, Muder R, Hoffman EL (2011). Increasing influenza and pneumococcal immunization rates in a nursing home network. J Am Med Dir Assoc.

[74] Rantz MJ, Zwygart-Stauffacher M, Hicks L, Mehr D, Flesner M, Petroski GF (2012). Randomized multilevel intervention to improve outcomes of residents in nursing homes in need of improvement. J Am Med Dir Assoc.

[75] Rantz MJ, Zwygart-Stauffacher M, Flesner M, Hicks L, Mehr D, Russell T (2012). Challenges of using quality improvement methods in nursing homes that “meed improvement”. J Am Med Dir Assoc.

[76] Rantz MJ, Zwygart-Stauffacher M, Flesner M, Hicks L, Mehr D, Russell T (2013). The influence of teams to sustain quality improvement in nursing homes that “need improvement”. J Am Med Dir Assoc.

[77] Seers K, Rycroft-Malone J, Cox K, Crichton N, Edwards RT, Eldh AC (2018). Facilitating implementation of research evidence (FIRE): an international cluster randomised controlled trial to evaluate two models of facilitation informed by the promoting action on research implementation in health services (PARIHS) framework. Implementation sci..

[78] Rycroft-Malone J, Seers K, Eldh AC, Cox K, Crichton N, Harvey G (2018). A realist process evaluation within the facilitating implementation of research evidence (FIRE) cluster randomised controlled international trial: an exemplar. Implementation sci..

[79] Tjia J, Field T, Mazor K, Lemay CA, Kanaan AO, Donovan JL (2015). Dissemination of evidence-based antipsychotic prescribing guidelines to nursing homes: a cluster randomized trial. J Am Geriatr Soc.

[80] Arling PA, Abrahamson K, Miech EJ, Inui TS, Arling G (2014). Communication and effectiveness in a US nursing home quality-improvement collaborative. Nurs Health Sci.

[81] Abrahamson K, Davila H, Mueller C, Inui T, Arling G (2013). Examining the lived experience of nursing home quality improvement: the case of a multifacility falls reduction project. J Gerontol Nurs.

[82] Azermai M, Wauters M, De Meester D, Renson L, Pauwels D, Peeters L (2017). A quality improvement initiative on the use of psychotropic drugs in nursing homes in Flanders. Acta Clin Belg.

[83] Hanson LC, Reynolds KS, Henderson M, Pickard CG (2005). A quality improvement intervention to increase palliative care in nursing homes. J Palliat Med.

[84] Jones KR, Fink R, Vojir C, Pepper G, Hutt E, Clark L (2004). Translation research in long-term care: improving pain management in nursing homes. Worldviews Evid-Based Nurs.

[85] Kaasalainen S, Ploeg J, Donald F, Coker E, Brazil K, Martin-Misener R (2015). Positioning clinical nurse specialists and nurse practitioners as change champions to implement a pain protocol in long-term care. Pain Manag Nurs.

[86] Olsho LEW, Spector WD, Williams CS, Rhodes W, Fink RV, Limcangco R (2014). Evaluation of AHRQ's on-time pressure ulcer prevention program: a facilitator-assisted clinical decision support intervention for nursing homes. Med Care.

[87] Rantz MJ, Popejoy L, Vogelsmeier A, Galambos C, Alexander G, Flesner M (2017). Successfully reducing hospitalizations of nursing home residents: results of the Missouri quality initiative. J Am Med Dir Assoc.

[88] Flesner M, Lueckenotte A, Vogelsmeier A, Popejoy L, Canada K, Minner D (2019). Advanced practice registered nurses' quality improvement efforts to reduce antipsychotic use in nursing homes. J Nurs Care Qual.

[89] Popejoy L, Vogelsmeier A, Galambos C, Flesner M, Alexander G, Lueckenotte A (2017). The APRN role in changing nursing home quality: the Missouri quality improvement initiative. J Nurs Care Qual.

[90] Vogelsmeier A, Popejoy L, Rantz M, Flesner M, Lueckenotte A, Alexander G (2015). Integrating advanced practice registered nurses into nursing homes: the Missouri quality initiative experience. J Nurs Care Qual.

[91] Rask K, Parmelee PA, Taylor JA, Green D, Brown H, Hawley J, et al. Implementation and evaluation of a nursing home fall management program 2007;55(3):342–349. 10.1111/j.1532-5415.2007.01083.x 17341235

[92] Sales AE, Schalm C, Baylon MA, Fraser KD (2014). Data for improvement and clinical excellence: report of an interrupted time series trial of feedback in long-term care. Implementation sci..

[93] Sales AE, Fraser K, Baylon MA, O'Rourke HM, Gao G, Bucknall T (2015). Understanding feedback report uptake: process evaluation findings from a 13-month feedback intervention in long-term care settings. Implementation sci..

[94] Abel RL, Warren K, Bean G, Gabbard B, Lyder CH, Bing M (2005). Quality improvement in nursing homes in Texas: results from a pressure ulcer prevention project. J Am Med Dir Assoc.

[95] Badger F, Clifford C, Hewison A, Thomas K (2009). An evaluation of the implementation of a programme to improve end-of-life care in nursing homes. Palliat Med.

[96] Baier RR, Gifford DR, Lyder CH, Schall MW, Funston-Dillon DL, Lewis JM (2003). Quality improvement for pressure ulcer care in the nursing home setting: the northeast pressure ulcer project. J Am Med Dir Assoc.

[97] Baier RR, Gifford DR, Patry G, Banks SM, Rochon T, DeSilva D (2004). Ameliorating pain in nursing homes: a collaborative quality-improvement project. J Am Geriatr Soc.

[98] Boyle PJ, O’Neil KW, Berry CA, Stowell SA, Miller SC (2013). Improving diabetes care and patient outcomes in skilled-care communities: successes and lessons from a quality improvement initiative. J Am Med Dir Assoc.

[99] Bravo G, Dubois MF, Roy PM (2005). Improving the quality of residential care using goal attainment scaling. J Am Med Dir Assoc.

[100] Buhr GT, White HK (2006). Quality improvement initiative for chronic pain assessment and management in the nursing home: a pilot study. J Am Med Dir Assoc.

[101] Carson J, Gottheil S, Gob A, Lawson S (2017). London transfer project: improving handover documentation from long-term care homes to hospital emergency departments. BMJ Open Qual.

[102] Chodosh J, Price RM, Cadogan MP, Damron-Rodriguez J, Osterweil D, Czerwinski A (2015). A practice improvement education program using a mentored approach to improve nursing facility depression care-preliminary data. J Am Geriatr Soc.

[103] Colon-Emeric C, Schenck A, Gorospe J, McArdle J, Dobson L, Deporter C (2006). Translating evidence-based falls prevention into clinical practice in nursing facilities: results and lessons from a quality improvement collaborative. J Am Geriatr Soc.

[104] Dolansky MA, Hitch JA, Piña IL, Boxer RS. Improving heart failure disease management in skilled nursing facilities: lessons learned. Clin Nurs Res. 2013;432–47. 10.1177/1054773813485088 23606187

[105] Edwards HE, Chang AM, Gibb M, Finlayson KJ, Parker C, O'Reilly M (2017). Reduced prevalence and severity of wounds following implementation of the champions for skin integrity model to facilitate uptake of evidence-based practice in aged care. J Clin Nurs.

[106] Fallon T, Buikstra E, Cameron M, Hegney D, Mackenzie D, March J (2006). Implementation of oral health recommendations into two residential aged care facilities in a regional Australian city. Int J Evid Based Healthc.

[107] Fine PG, Bradshaw DH, Cohen MJ, Connor SR, Donaldson G, Gharibo C (2014). Evaluation of the performance improvement CME paradigm for pain management in the long-term care setting. Pain Med.

[108] Hickman SE, Unroe KT, Ersek MT, Buente B, Nazir A, Sachs GA (2016). An interim analysis of an advance care planning intervention in the nursing home setting. J Am Geriatr Soc.

[109] Ersek M, Hickman SE, Thomas AC, Bernard B, Unroe KT (2018). Stakeholder perspectives on the optimizing patient transfers, impacting medical quality, and improving iymptoms: transforming institutional care (OPTIMISTIC) project. Gerontologist..

[110] Horn SD, Sharkey SS, Hudak S, Gassaway J, James R, Spector W (2010). Pressure ulcer prevention in long-term-care facilities: a pilot study implementing standardized nurse aide documentation and feedback reports. Adv Skin Wound Care.

[111] Sharkey S, Hudak S, Horn SD, Barrett R, Spector W, Limcangco R. Exploratory study of nursing home factors associated with successful implementation of clinical decision support tools for pressure ulcer prevention. Adv Skin Wound Care. 2013;26(2):83–92; quiz p.3. 10.1097/01.ASW.0000426718.59326.bb 23337649

[112] Horner JK, Hanson LC, Wood D, Silver AG, Reynolds KS (2005). Using quality improvement to address pain management practices in nursing homes. J Pain Symptom Manag.

[113] Keeney CE, Scharfenberger JA, O'Brien JG, Looney S, Pfeifer MP, Hermann CP (2008). Initiating and sustaining a standardized pain management program in long-term care facilities. J Am Med Dir Assoc.

[114] Kezirian AC, McGregor MJ, Stead U, Sakaluk T, Spring B, Turgeon S (2018). Advance care planning in the nursing home setting: a practice improvement evaluation. J Soc Work End Life Palliat Care.

[115] Kovach CR, Morgan S, Noonan PE, Brondino M (2008). Using principles of diffusion of innovation to improve nursing home care. J Nurs Care Qua.

[116] Lai CC, Lu MC, Tang HJ, Chen YH, Wu YH, Chiang HT (2018). Implementation of a national quality improvement program to enhance hand hygiene in nursing homes in Taiwan. J Microbiol Immunol Infect.

[117] Lynn J, West J, Hausmann S, Gifford D, Nelson R, McGann P (2007). Collaborative clinical quality improvement for pressure ulcers in nursing homes. J Am Geriatr Soc.

[118] Ouslander JG, Perloe M, Givens JH, Kluge L, Rutland T, Lamb G (2009). Reducing potentially avoidable hospitalizations of nursing home residents: results of a pilot quality improvement project. J Am Med Dir Assoc.

[119] Rosen J, Mittal V, Degenholtz H, Castle N, Mulsant B, Rhee YJ (2005). Organizational change and quality improvement in nursing homes: approaching success. J Healthc Qual.

[120] Rosen J, Mittal V, Degenholtz H, Castle N, Mulsant BH, Nace D (2006). Pressure ulcer prevention in black and white nursing home residents: a QI initiative of enhanced ability, incentives, and management feedback. Adv Skin Wound Care.

[121] Scott-Cawiezell J, Madsen RW, Pepper GA, Vogelsmeier A, Petroski G, Zellmer D (2009). Medication safety teams' guided implementation of electronic medication administration records in five nursing homes. Jt Comm J Qual Patient Saf.

[122] Simmons SF, Durkin DW, Shotwell MS, Erwin S, Schnelle JF (2013). A staff training and management intervention in VA long-term care: impact on feeding assistance care quality. Transl Behav Med.

[123] Tena-Nelson R, Santos K, Weingast E, Amrhein S, Ouslander J, Boockvar K (2012). Reducing potentially preventable hospital transfers: results from a thirty nursing home collaborative. J Am Med Dir Assoc.

[124] Törmä J, Winblad U, Saletti A, Cederholm T (2014). Strategies to implement community guidelines on nutrition and their long-term clinical effects in nursing home residents. J Nutr Health Aging.

[125] Triller D, Wymer S, Morris K, Farman G, Myrka A (2014). Improving warfarin safety in long-term care. Consult Pharm.

[126] Wilson J, Bak A, Tingle A, Greene C, Tsiami A, Canning D (2018). Improving hydration of care home residents by increasing choice and opportunity to drink: a quality improvement study. Clin Nutr.

[127] Zubkoff L, Neily J, Quigley P, Delanko V, Young-Xu Y, Boar S (2018). Preventing falls and fall-related injuries in state veterans homes: virtual breakthrough series collaborative. J Nurs Care Qual.

