# Supplementary material for: Quality improvement studies in nursing homes: a scoping review
Source: BMC Health Serv Res. 2021 Aug 12;21:803. doi: 10.1186/s12913-021-06803-8 (PMC8361800; doi:10.1186/s12913-021-06803-8)
Supplement: Supplementary file 1 — Additional file 1. [file 12913_2021_6803_MOESM1_ESM.docx]

**Additional File 1**

**Authors**

Mark Toles, University of North Carolina at Chapel Hill

Cathleen Colón-Emeric, Duke University and Durham VA GRECC

Elizabeth Moreton, University of North Carolina at Chapel Hill

Lauren Frey, University of North Carolina at Chapel Hill

Jennifer Leeman, University of North Carolina at Chapel Hill

**Searches conducted in July 2018 and updated Feb. 28, 2019**

**PubMed (ncbi.nlm.nih.gov)**

Note: This search was conducted in the legacy version of PubMed which will be retired 10/31/2020

("Residential Facilities"[Mesh] OR "Assisted Living Facilities"[Mesh] OR "Homes for the Aged"[Mesh] OR "Nursing Homes"[Mesh] OR "nursing home"[title/abstract] OR "nursing homes"[title/abstract]  OR "care home"[title/abstract] OR "care homes"[title/abstract] OR "residential care"[title/abstract] OR "continuing care"[title/abstract] OR "assisted living"[title/abstract] OR "long term care"[title/abstract] OR "skilled nursing"[title/abstract] OR SNF[title/abstract] OR “extended care”[title/abstract] OR “homes for the aged”[title/abstract] OR “home for the aged”[title/abstract] OR “housing for the elderly”[title/abstract] OR “old people home”[title/abstract] OR “old people homes”[title/abstract] OR “old age homes”[title/abstract] OR “old age home”[title/abstract] OR “aged care home”[title/abstract] OR “aged care homes”[title/abstract] OR “aged care facility”[title/abstract] OR “aged care facilities”[title/abstract] OR “convalescence home”[title/abstract] OR “convalescence homes”[title/abstract] OR “convalescence hospital”[title/abstract] OR “convalescence hospitals”[title/abstract]) AND (QAPI OR "continuous improvement"[title/abstract] OR "quality assurance"[title/abstract] OR "lean methodology"[title/abstract] OR "lean manufacturing"[title/abstract] OR "lean production"[title/abstract] OR "lean healthcare"[title/abstract] OR "lean thinking"[title/abstract] OR "lean six sigma"[title/abstract] OR A3[title/abstract] OR "value stream"[title/abstract] OR 5S[title/abstract] OR "five S"[title/abstract] OR kanban[title/abstract] OR "poka-yoke"[title/abstract] OR "total productive maintenance"[title/abstract] OR "just-in-time"[title/abstract] OR muda[title/abstract] OR muri[title/abstract] OR mura[title/abstract] OR "six sigma"[title/abstract] OR kaizen[title/abstract] OR 6σ[title/abstract] OR "quality improvement"[title/abstract] OR "quality management"[title/abstract] OR "quality planning"[title/abstract] OR "process improvement"[title/abstract] OR "process capability"[title/abstract] OR "performance improvement"[title/abstract] OR "quality function deployment"[title/abstract] OR QFD[title/abstract] OR SIPOC[title/abstract] OR "failure mode and effects analysis"[title/abstract] OR FMEA[title/abstract] OR "plan do check act"[title/abstract]  OR PDCA[title/abstract] OR “plan do study act”[title/abstract] OR PDSA[title/abstract] OR "root cause analysis"[title/abstract] OR "five whys"[title/abstract] OR "fishbone diagram"[title/abstract] OR "5 whys"[title/abstract] OR DMAIC[title/abstract] OR DMADV[title/abstract] OR DFSS[title/abstract] OR "loss function"[title/abstract] OR deming[title/abstract] OR taguchi[title/abstract] OR ishikawa[title/abstract]) AND ( "2003/01/01"[PDat] : "3000/12/31"[PDat] ) AND English[lang] NOT (Addresses[Publication Type] OR Autobiography[Publication Type] OR Bibliography[Publication Type] OR Biography[Publication Type] OR Books and Documents[Publication Type] OR Classical Article[Publication Type] OR Clinical Conference[Publication Type] OR Comment[Publication Type] OR Congresses[Publication Type] OR Consensus Development Conference[Publication Type] OR Consensus Development Conference, NIH[Publication Type] OR Dataset[Publication Type] OR Dictionary[Publication Type] OR Directory[Publication Type] OR Duplicate Publication[Publication Type] OR Editorial[Publication Type] OR Electronic Supplementary Materials[Publication Type] OR Festschrift[Publication Type] OR Government Publications[Publication Type] OR Historical Article[Publication Type] OR Interactive Tutorial[Publication Type] OR Interview[Publication Type] OR Introductory Journal Article[Publication Type] OR Lectures[Publication Type] OR Legal Cases[Publication Type] OR Legislation[Publication Type] OR Letter[Publication Type] OR News[Publication Type] OR Newspaper Article[Publication Type] OR Overall[Publication Type] OR Patient Education Handout[Publication Type] OR Periodical Index[Publication Type] OR Personal Narratives[Publication Type] OR Portraits[Publication Type] OR Retracted Publication[Publication Type] OR Retraction of Publication[Publication Type] OR Scientific Integrity Review[Publication Type] OR Technical Report[Publication Type] OR Video-Audio Media[Publication Type] OR Webcasts[Publication Type])

**CINAHL Plus with Full Text (Ebsco)**

((MH "Residential Facilities") OR (MH "Nursing Homes+") OR (MH "Housing for the Elderly") OR TI("nursing home" OR "nursing homes"  OR "care home" OR "care homes" OR "residential care" OR "continuing care" OR "assisted living" OR "long term care" OR "skilled nursing" OR SNF OR “extended care” OR “homes for the aged” OR “home for the aged” OR “housing for the elderly” OR “old people home” OR “old people homes” OR “old age homes” OR “old age home” OR “aged care home” OR “aged care homes” OR “aged care facility” OR “aged care facilities” OR “convalescence home” OR “convalescence homes” OR “convalescence hospital” OR “convalescence hospitals”) OR AB("nursing home" OR "nursing homes"  OR "care home" OR "care homes" OR "residential care" OR "continuing care" OR "assisted living" OR "long term care" OR "skilled nursing" OR SNF OR “extended care” OR “homes for the aged” OR “home for the aged” OR “housing for the elderly” OR “old people home” OR “old people homes” OR “old age homes” OR “old age home” OR “aged care home” OR “aged care homes” OR “aged care facility” OR “aged care facilities” OR “convalescence home” OR “convalescence homes” OR “convalescence hospital” OR “convalescence hospitals”)) AND (QAPI OR (TI(‘continuous improvement’ OR ‘quality assurance’ OR ‘lean methodology’ OR ‘lean manufacturing’ OR ‘lean production’ OR ‘lean healthcare’ OR ‘lean thinking’ OR ‘lean six sigma’ OR A3 OR ‘value stream’ OR 5S OR ‘five S’ OR kanban OR ‘poka-yoke’ OR ‘total productive maintenance’ OR ‘just-in-time’ OR muda OR muri OR mura OR ‘six sigma’ OR kaizen OR 6σ OR ‘quality improvement’ OR ‘quality management’ OR ‘quality planning’ OR ‘process improvement’ OR ‘process capability’ OR ‘performance improvement’ OR ‘quality function deployment’ OR QFD OR SIPOC OR ‘failure mode and effects analysis’ OR FMEA OR ‘plan do check act’  OR PDCA OR ‘plan do study act’ OR PDSA OR ‘root cause analysis’ OR ‘five whys’ OR ‘fishbone diagram’ OR ‘5 whys’ OR DMAIC OR DMADV OR DFSS OR ‘loss function’ OR deming OR taguchi OR ishikawa) OR AB(‘continuous improvement’ OR ‘quality assurance’ OR ‘lean methodology’ OR ‘lean manufacturing’ OR ‘lean production’ OR ‘lean healthcare’ OR ‘lean thinking’ OR ‘lean six sigma’ OR A3 OR ‘value stream’ OR 5S OR ‘five S’ OR kanban OR ‘poka-yoke’ OR ‘total productive maintenance’ OR ‘just-in-time’ OR muda OR muri OR mura OR ‘six sigma’ OR kaizen OR 6σ OR ‘quality improvement’ OR ‘quality management’ OR ‘quality planning’ OR ‘process improvement’ OR ‘process capability’ OR ‘performance improvement’ OR ‘quality function deployment’ OR QFD OR SIPOC OR ‘failure mode and effects analysis’ OR FMEA OR ‘plan do check act’  OR PDCA OR ‘plan do study act’ OR PDSA OR ‘root cause analysis’ OR ‘five whys’ OR ‘fishbone diagram’ OR ‘5 whys’ OR DMAIC OR DMADV OR DFSS OR ‘loss function’ OR deming OR taguchi OR ishikawa))) AND LA(English) AND DT 2003-2019 NOT (PT Algorithm OR PT Anecdote OR PT Bibliography OR PT Biography OR PT Book OR PT Book Chapter OR PT Book Review OR PT Brief Item OR PT Care Plan OR PT Cartoon OR PT CEU OR PT Code of Ethics OR PT Commentary OR PT Computer Program OR PT Consumer/Patient Teaching Materials OR PT Critical Path OR PT Diagnostic Images OR PT Directories OR PT Doctoral Dissertation OR PT Editorial OR PT Equations & Formulas OR PT Evidence-Based Care Sheet OR PT Exam Questions OR PT Forms OR PT Games OR PT Glossary OR PT Historical Material OR PT Interview OR PT Legal Case OR PT Letter OR PT Masters Thesis OR PT Nurse Practice Acts OR PT Nursing Diagnoses OR PT Obituary OR PT Pamphlet OR PT Pamphlet Chapter OR PT Pictorial OR PT Poetry OR PT Practice Acts OR PT Proceedings OR PT Protocol OR PT Questionnaire/Scale OR PT Questions and Answers OR PT Quick Lesson OR PT Research Instrument OR PT Response OR PT Standards OR PT Statistics OR PT Tables/Charts OR PT Teaching Materials OR PT Tracings OR PT Website )

**Embase (Embase.com)**

('nursing home'/exp OR 'nursing homes'/exp OR 'residential care'/exp OR 'residential facilities'/exp OR 'assisted living'/exp OR 'skilled nursing facility'/exp OR 'long term care facility'/exp OR 'extended care facility'/exp OR 'homes for the aged'/exp OR 'home for the aged'/exp OR 'housing for the elderly'/exp OR 'old people home'/exp OR 'old age homes'/exp OR 'old age home'/exp OR 'convalescence home'/exp OR 'convalescence hospital'/exp OR 'nursing home':ti,ab OR 'nursing homes':ti,ab OR 'care home':ti,ab OR 'care homes':ti,ab OR 'residential care home':ti,ab OR 'residential care facility':ti,ab OR 'residential care facilities':ti,ab OR 'continuing care':ti,ab OR 'assisted living':ti,ab OR 'long term care facility':ti,ab OR 'long term care facilities':ti,ab OR 'skilled nursing':ti,ab OR snf:ti,ab OR 'extended care facility':ti,ab OR 'extended care facilities':ti,ab OR 'homes for the aged':ti,ab OR 'home for the aged':ti,ab OR 'housing for the elderly':ti,ab OR 'old people home':ti,ab OR 'old people homes':ti,ab OR 'old age homes':ti,ab OR 'old age home':ti,ab OR 'aged care home':ti,ab OR 'aged care homes':ti,ab OR 'aged care facility':ti,ab OR 'aged care facilities':ti,ab OR 'convalescence home':ti,ab OR 'convalescence homes':ti,ab OR 'convalescence hospital':ti,ab OR 'convalescence hospitals':ti,ab) AND (QAPI OR 'quality control'/de OR ‘benchmarking’/exp OR ‘clinical audit’/exp OR ‘instrument validation’/exp OR ‘nursing audit’/exp OR ‘quality circle’/exp OR ‘total quality management’/exp OR ‘validation process’/exp OR 'lean six sigma'/exp OR 'performance improvement'/exp OR 'quality function deployment'/exp OR 'failure mode and effects analysis'/exp OR 'root cause analysis'/exp OR ‘continuous improvement’:ti,ab OR ‘quality assurance’:ti,ab OR ‘lean methodology’:ti,ab OR ‘lean manufacturing’:ti,ab OR ‘lean production’:ti,ab OR ‘lean healthcare’:ti,ab OR ‘lean thinking’:ti,ab OR ‘lean six sigma’:ti,ab OR A3:ti,ab OR ‘value stream’:ti,ab OR 5S:ti,ab OR ‘five S’:ti,ab OR kanban:ti,ab OR ‘poka-yoke’:ti,ab OR ‘total productive maintenance’:ti,ab OR ‘just-in-time’:ti,ab OR muda:ti,ab OR muri:ti,ab OR mura:ti,ab OR ‘six sigma’:ti,ab OR kaizen:ti,ab OR 6σ:ti,ab OR ‘quality improvement’:ti,ab OR ‘quality management’:ti,ab OR ‘quality planning’:ti,ab OR ‘process improvement’:ti,ab OR ‘process capability’:ti,ab OR ‘performance improvement’:ti,ab OR ‘quality function deployment’:ti,ab OR QFD:ti,ab OR SIPOC:ti,ab OR ‘failure mode and effects analysis’:ti,ab OR FMEA:ti,ab OR ‘plan do check act’:ti,ab  OR PDCA:ti,ab OR ‘plan do study act’:ti,ab OR PDSA:ti,ab OR ‘root cause analysis’:ti,ab OR ‘five whys’:ti,ab OR ‘fishbone diagram’:ti,ab OR ‘5 whys’:ti,ab OR DMAIC:ti,ab OR DMADV:ti,ab OR DFSS:ti,ab OR ‘loss function’:ti,ab OR deming:ti,ab OR taguchi:ti,ab OR ishikawa:ti,ab OR 'health care quality'/de) AND [english]/lim AND [2003-2019]/py NOT ([conference abstract]/lim OR [conference review]/lim OR [editorial]/lim OR [erratum]/lim OR [letter]/lim OR [note]/lim OR [short survey]/lim)
