# Supplementary material for: Quality improvement studies in nursing homes: a scoping review
Source: BMC Health Serv Res. 2021 Aug 12;21:803. doi: 10.1186/s12913-021-06803-8 (PMC8361800; doi:10.1186/s12913-021-06803-8)
Supplement: Supplementary file 2 — Additional file 2. [file 12913_2021_6803_MOESM2_ESM.docx]

**Additional File 2**

**Authors**

Mark Toles, University of North Carolina at Chapel Hill

Cathleen Colón-Emeric, Duke University and Durham VA GRECC

Elizabeth Moreton, University of North Carolina at Chapel Hill

Lauren Frey, University of North Carolina at Chapel Hill

Jennifer Leeman, University of North Carolina at Chapel Hill

**[next page]**

**PRISMA-ScR Diagram**

Records identified through database searching
PubMed = 914

Embase = 1807

CINAHL = 533

(n = 3254)

Records after duplicates removed
(n = 2302)

Full-text articles assessed for eligibility
(n = 98)

Records screened
(n = 2302)

Full-text articles excluded
(n = 21)

- Less than two nursing homes = 11
- Not in nursing homes = 5
- Not quality improvement = 2
- No service or resident outcome = 3

Records excluded
(n = 2204)

Additional records identified through other sources
(n = 5)

Articles included in qualitative synthesis
(n = 77)
